# Contactless Simultaneous Breathing and Heart Rate Detections in Physical Activity Using IR-UWB Radars

**DOI:** 10.3390/s21165503

**Published:** 2021-08-16

**Authors:** Xinyue Zhang, Xiuzhu Yang, Yi Ding, Yili Wang, Jialin Zhou, Lin Zhang

**Affiliations:** 1 School of Artificial Intelligence, Beijing University of Posts and Telecommunications, Beijing 100876, China; zhangxinyue2019@bupt.edu.cn (X.Z.); yangxiuzhu@bupt.edu.cn (X.Y.); dingyi97@bupt.edu.cn (Y.D.); fanshionline@bupt.edu.cn (Y.W.); zhoujialin@bupt.edu.cn (J.Z.); 2 School of Informations Communication Engineering, Beijing Information Science and Technology University, Beijing 100192, China

**Keywords:** IR-UWB radar, vital signs, physical activity, movement interference alleviation

## Abstract

Vital signs monitoring in physical activity (PA) is of great significance in daily healthcare. Impulse Radio Ultra-WideBand (IR-UWB) radar provides a contactless vital signs detection approach with advantages in range resolution and penetration. Several researches have verified the feasibility of IR-UWB radar monitoring when the target keeps still. However, various body movements are induced by PA, which lead to severe signal distortion and interfere vital signs extraction. To address this challenge, a novel joint chest–abdomen cardiopulmonary signal estimation approach is proposed to detect breath and heartbeat simultaneously using IR-UWB radars. The movements of target chest and abdomen are detected by two IR-UWB radars, respectively. Considering the signal overlapping of vital signs and body motion artifacts, Empirical Wavelet Transform (EWT) is applied on received radar signals to remove clutter and mitigate movement interference. Moreover, improved EWT with frequency segmentation refinement is applied on each radar to decompose vital signals of target chest and abdomen to vital sign-related sub-signals, respectively. After that, based on the thoracoabdominal movement correlation, cross-correlation functions are calculated among chest and abdomen sub-signals to estimate breath and heartbeat. The experiments are conducted under three kinds of PA situations and two general body movements, the results of which indicate the effectiveness and superiority of the proposed approach.

## 1. Introduction

Physical activity (PA) is of great significance in daily healthcare; it improves the level of both mental and physiological health and reduces the risk of several medical illnesses. It is reported that the ratio of sudden cardiac death is approximately 1 in 50,000 athlete-years for the young athletes, and even reaches 1 in 5000 for risky groups [1]. Long-term monitoring of vital signs, e.g., heartbeat and breath, helps to prevent sport injury and improve the effectiveness of PA.

Conventional vital signs monitoring methods during exercising are based on wearable devices, such as plethysmography (PPG) sensors [2] and piezoresistive sensors [3]. These devices are attached to human body directly, which easily lead to body swelling and discomfort when wearing overtime. Contactless vital signs monitoring is a new research trend in fitness healthcare. There exist several non-contact solutions in PA scenarios. Vision-based sensors are applied to obtain remote-plethysmography (rPPG) signal reflected from human face for heartbeat extraction [4]. Nevertheless, the usage of vision-based sensors is strictly limited by lighting condition, let alone the inevitable privacy issues. Thermal-based sensors provide another solution for physiological signal detection and analysis [5]. Furthermore, variation of environment temperature hinders the monitoring performance. Radar-based methods deal with aforementioned challenges effectively.

When the target keeps still, many remote sensing solutions are provided based on various radar systems, mainly consisting of the Continuous Wave (CW) radar [6,7], Frequency Modulated Continuous Wave (FMCW) radar [8,9,10,11], and Impulse Radio Ultra-WideBand (IR-UWB) radar [12,13,14,15,16,17]. CW radar is not only hindered by the calibration difficulty caused by the null point issue, but also lacks ability of range detection, which brings challenge for vital signs monitoring during PA. Moreover, compared with FMCW radar, IR-UWB radar has advantages in range resolution and interference-resistance performances [18]. Moreover, the low power consumption of the IR-UWB radar is promising for continues vital signs monitoring. Besides, compared with existing Multiple-Input Multiple-Output (MIMO) radar systems [19,20] and self-motion radar systems [21,22], the monostatic IR-UWB radar deployed at a fixed location has low system complexity without extra self-movement interference during detection, which is suitable for monitoring weak physiological signals during PA.

As is demonstrated above, most of the existing radar-based researches estimate vital signs by detecting chest vibration induced by cardiopulmonary movement [13,14,15,16,17]. Due to the physical structure of human body, human breath movement leads to relative vibrations of both chest and abdomen. It is proved that the abdomen fluctuation is much stronger than the chest vibration induced by breath [23], so that several researches attempt to monitor breath for abdomen movement [24,25]. Based on the thoracoabdominal vibration association caused by human breath, there exist researches detecting both chest and abdominal movement simultaneously to recognize respiration pattern [26], diagnose sleep apnea–hypopnea syndrome [27], and track tumors based on breathing motions [28]. Nevertheless, these researches analyze physiological signals when the participants keep still without large motions. During PA, the weak vital signal is distorted and overlapped by unexpected body movement interference, which severely hinders the vital signs extraction.

In order to mitigate the effect of body movement on vital signs estimation performance, several researches stop monitoring when large body motion is detected [29,30]. Moreover, there exist researches trying to mitigate motion artifacts and monitor vital signs during target moving. The work in [31] deals with random body movements artifacts while driving and detects vital signs by segmenting and reconstructing vital signal based on the correlation method. The authors of [32,33,34] monitor heartbeat and breath with the target swing body back and forth. The work in [32] develops an IR-UWB radar system with MIMO prototype to detect thorax signals from both target front and the back, and an iterative adaptive approach is applied to determine respiration and heartbeat rates. The work in [33] presents a multi-feature alignment two-layer EEMD method to detect breath and heart rate to alleviate body fluctuation interference in the driving scenario. The work in [34] introduces a motion-tolerant vital signs detection method using two radars towards to target chest. In our previous work, a Heartbeat Estimation And Recovery (HEAR) method using an IR-UWB radar is proposed to alleviate interference caused by large body movement and estimate vital signs during the target walking [35]. However, during PA, not only a single type motion artifact mentioned above is induced, such as the variation of body-to-radar distance or random body movement, but various motions of different body parts are contained including arm waving, waist twisting, and body moving. It is challenging to cope with breath and heartbeat detection with such motion interference using the existing methods.

**Figure 1:**
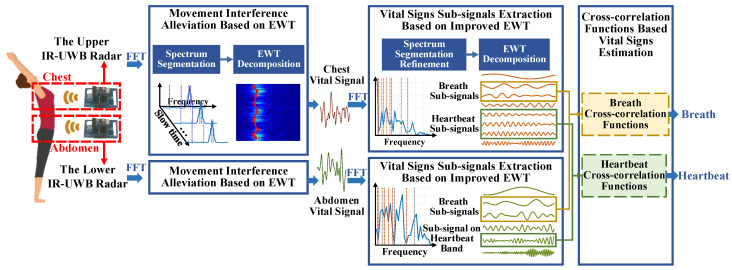
Flowchart of the joint chest–abdomen cardiopulmonary signal estimation approach.

In this paper, a novel joint chest–abdomen cardiopulmonary signal estimation approach is proposed to detect breath and heartbeat simultaneously using IR-UWB radars during PA. The flowchart of the proposed approach is depicted in Figure 1. Two radars are deployed to monitor the movement of chest and abdomen synchronously to collect target signals containing vital signs. During PA, the vital signals are overlapped and distorted by motion artifacts from various body parts. In order to remove clutter and mitigate motion interference, Empirical Wavelet Transform (EWT) is applied on received radar signals. Moreover, improved EWT with refined frequency segmentation decomposes target chest and abdomen vital signals obtained from two radars, respectively, and the breath and heartbeat related sub-signals are extracted. After that, cross-correlation functions are calculated among chest and abdomen sub-signals to extract heartbeat and breath simultaneously. Experiments are conducted in three different PA scenarios including a set of gentle yoga movement and two daily exercises, and two general body movements including random body movement and back-and-forth body movement. The experiment results demonstrate the effectiveness and robustness of the proposed approach.

The three main contributions of this paper are summarized as follows:

- In order to remove background clutters and alleviate movement interference, EWT is introduced to decompose received radar signals and reduce motion artifacts from various body parts.
- In order to further extract breath and heartbeat related signals, improved EWT with frequency segmentation refinement is applied on each radar to decompose vital signals of the chest and abdomen, respectively. Thus, vital signs sub-signals are obtained.
- In order to estimate breath and heartbeat under the interference of multi-order harmonics and residual motion artifacts, the cross-correlation functions are calculated among chest and abdomen sub-signals. Based on the thoracoabdominal correlation, the heartbeat and breath are extracted simultaneously.

The rest of this paper is organized as follows. Section 2 describes the experimental setup. Section 3 introduces the proposed approach. Section 4 presents the experimental results and discussion, and Section 5 concludes this paper.

## 2. Experimental Setup

**Figure 2:**
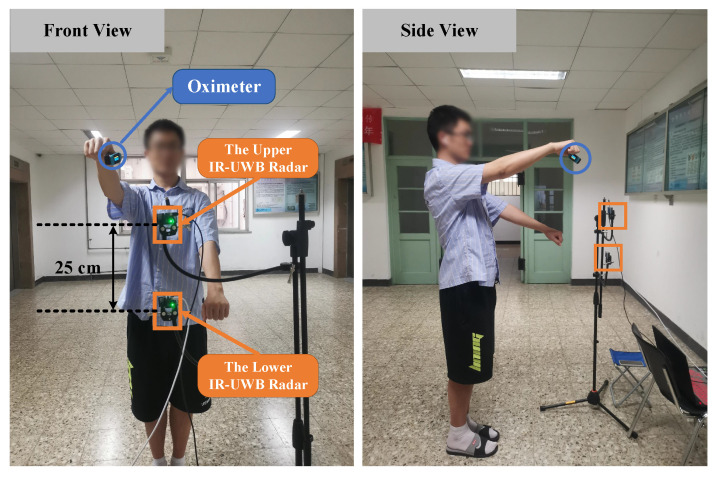
Experiment setup.

As is shown in Figure 2, the experiments are conducted indoors to evaluate the proposed approach. Two Novelda X4M03 IR-UWB radars are deployed to collect target signals, which are based on a complete X4 UWB radar System of Chip (SoC) [36]. The sampling rate is 23.328 GS/s, resulting in a high range resolution of ~6.5 millimeters. The carrier frequency is 7.25 GHz with 2.5 GHz working band, which falls in the UWB bandwidth limitation specified by the Federal Communications Commission (FCC). Moreover, the radar pulses working in this frequency band are allowed to penetrate clothes and detect human skin vibrations. A pair of antennas with beamwidth about 60° in both azimuth and elevation are integrated with the radar SoC on the Printed Circuit Board (PCB). Besides, the transmission power maintains low-power emission below −41.3 dBm/MHz, which does not harm human health for long-term vital signs monitoring. The maximum detection range of the radar is set to 2 meters with 20 frames collection per second.

**Figure 3:**
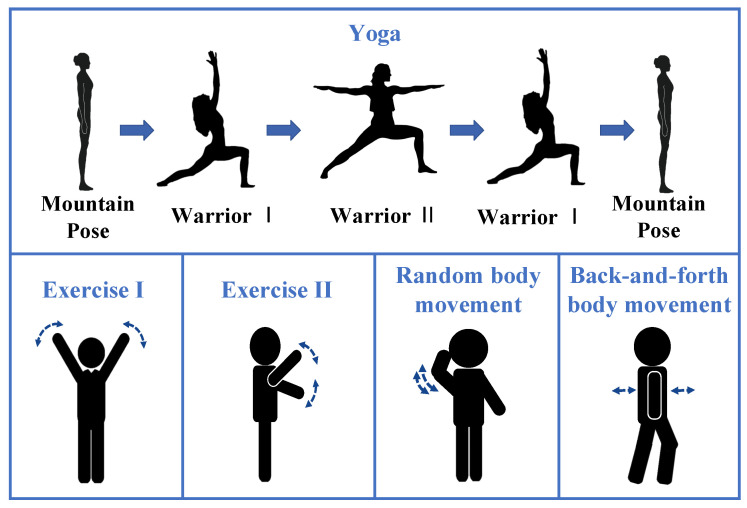
Five kinds of activities.

Both radars are placed in front of the human body along the center line of the human trunk. As is illustrated in Figure 3, the upper IR-UWB radar is placed about the chest height, while the lower IR-UWB radar is placed about the abdomen height of a normal adult. Based on the human body structure, the height difference of these two radars is set to 25 cm so that it is feasible to detect the vibrations of chest and abdomen in the effective detection angle range of antennas. Both of them are controlled by a laptop to collect and transmit the radar data through USB ports synchronously. All the collected data are processed further on a laptop with an Intel’s Core i5 10th Generation processor.

As is presented in Figure 3, a total of 5 kinds of activities are performed in the experiment, including three kinds of PA, i.e., a set of gentle yoga and two daily exercises, and two general body movements, i.e., random body movement and back-and-forth body movement. The details of body motions are listed in Table 1. During data collection, the participants are required to stay relax, breath evenly, and keep the corresponding movement following the guidance. An oximeter with medical device certificate provides instantaneous heart rates and breath rate as references. The heartbeat is extracted based on the plethysmogram signal, whereas the continues breath rate is detected based on the oxygen saturation variation. A total of 8 participants with different genders, ages, and body shapes are tested on these five kinds of activities. The detailed physical information of participants is listed in Table 2. A total of 73 records are collected, which are segmented by a fixed 15 s window with a step of 2 s sliding window. A total of 3869 slices are obtained.

## 3. Methods

### 3.1. IR-UWB Signal Model and Preprocessing

Based on the radar time of arrival model, the propagation time of received signal reflected from distance *d* is expressed as

(1) $$\tau \left(t\right)=\frac{2d\left(t\right)}{c},$$

where τ denotes the signal propagation time regarded as radar fast time, *t* denotes the radar observation time regarded as radar slow time, and *c* denotes the speed of light.

The received IR-UWB radar signal is first preprocessed to remove static background echoes by subtracting the average along the fast time. Then, the signal reflected from human body is obtained. Due to target movement during PA and different radar deployment positions, the target signal is reflected from various body parts such as thorax, limbs, and abdomen. The signal s(t,τ) reflected from target is obtained, which is modeled as

(2) $$s\left(t,\tau \right)=\sum_{n=1}^{K}p_{n}\left(,\tau \right)+p_{v}\left(t,\tau \right),$$

where $p_{v}\left(t,\tau \right)$ indicates the pulse from the interested part, that is, target chest for the upper radar or from target abdomen for the lower radar. $p_{n}\left(t,\tau \right)$ indicates the reflected signal from the other parts of the human body. *K* denotes the number of pulse channels. Considering the multi-order vital signs harmonics, the vibration of chest caused by breath is modeled as a summation of various sinusoidal waves, whereas the heartbeat is modeled as a summation of pulses [37]. The chest movement $\tau_{chest}\left(t\right)$ is described as

(3) $$\tau_{chest}\left(t\right)=\tau_{1}+\sum_{i}a_{h}^{i}\rho_{h}\left(t\right)+\sum_{j}a_{b}^{j}\sin\left(jf_{b}t+\varphi_{b_{1}}\right),$$

where $\tau_{1}$ indicates antenna-to-chest distance. $f_{b}$ denotes the breath frequency, while $\varphi_{b_{1}}$ denotes the phase of breath on target chest. $\rho_{h}\left(t\right)$ denotes the heart vibration pulse signal. $a_{h}^{i}$ and $a_{b}^{j}$ denote the amplitudes of vibrations.

Considering that the breath movement leads to vibrations of both chest and abdomen, the abdomen movement $\tau_{abdomen}\left(t\right)$ is described in the same way as

(4) $$\tau_{abdomen}\left(t\right)=\tau_{2}+\sum_{k}a_{b}^{k}\sin\left(kf_{b}t+\varphi_{b_{2}}\right),$$

where $\tau_{2}$ indicates antenna-to-abdomen distance, $a_{b}^{k}$ denotes the vibration amplitude, and $\varphi_{b_{2}}$ denotes the phase of breath on target abdomen.

In the paper, the two IR-UWB radars are deployed around target chest and abdomen, respectively. It is clarified that the received signal power attenuates severely along the increase of target-to-antenna angle [38] so that reflected signals from target chest and abdomen are relatively stronger, which can be extracted adequately. However, unlike scenarios where the target keeps still, there are various body motions during PA, which results in $\tau_{1}$ and $\tau_{2}$ varying along radar slow time. Moreover, the vital-signs-related signals are distorted and polluted by various body movements. It is infeasible to extract vital signs on a constant fast time directly. Moreover, the motion artifacts need to be alleviated to improve the performance of vital signal extraction. In order to obtain the varying $\tau_{chest}\left(t\right)$ and $\tau_{abdomen}\left(t\right)$ and estimate vital signs, EWT is introduced to mitigate movement interference. And the vital-signs-related sub-signals are further extracted.

### 3.2. Movement Interference Alleviation Based on EWT

EWT decomposes non-stationary signal into several sub-signals using a series of wavelet filters based on Fourier spectrum segments [39], which are applied to remove noise and extract interested signals in several researches [40,41,42]. After DC removal, there still exist clutters reflected from background. Moreover, due to the movement of multiple body parts during PA, the signals reflected from human body perform various frequency distribution in the Fourier spectrum so that EWT is applied on radar fast time to remove clutters and suppressing movement interference by decomposing radar signals.

The segmentation strategy based on local maxima and minima is introduced to obtain several narrow frequency sub-bands. The fast time frequency spectrum is obtained by Fast Fourier Transform (FFT). After that, all the local maxima are determined. The number of the Fourier segments *N* is set as

(5) $$N=\min\left\{M+1,N_{0}\right\},$$

where $N_{0}$ denotes the expected number of the Fourier segments, and *M* denotes the total number of local maxima. The N−1 largest local maxima are selected. Moreover, the positions of the lowest minimum are determined among consecutive selected maxima, which are regarded as the frequency boundaries to segment spectrum. Therefore, a total of *N* Fourier segments are obtained.

Based on the obtained frequency boundaries, a set of empirical wavelets is constructed as $\left\{\phi_{0}\left(\tau \right),{\left\{\psi_{q}\left(\tau \right)\right\}}_{i=1}^{N}\right\}$, which composes of a scaling function $\phi_{0}\left(\tau \right)$ and a series of empirical wavelets ${\left\{\psi_{q}\left(\tau \right)\right\}}_{i=1}^{N}$. Moreover, the signal is further decomposed as

(6) $$T_{s}\left(q,\tau \right)=\langle s,\psi_{q}\rangle =\int s\left(\upsilon \right)\bar{\psi_{q}\left(\upsilon -\tau \right)}d\upsilon ,$$

(7) $$T_{s}\left(0,\tau \right)=\langle s,\phi_{0}\rangle =\int s\left(\upsilon \right)\bar{\phi_{0}\left(\upsilon -\tau \right)}d\upsilon ,$$

where $T_{s}\left(q,\tau \right)$ and $T_{s}\left(0,\tau \right)$ indicate the detailed coefficient and the approximation coefficient, respectively. The overlines of $\psi_{q}$ and $\phi_{0}$ represent conjugate. Then the sub-signals, $s_{0}$ and $s_{q}$, are reconstructed as

(8) $$s_{0}\left(\tau \right)=T_{s}\left(0,\tau \right)*\phi_{0}\left(\tau \right)$$

(9) $$s_{q}\left(\tau \right)=T_{s}\left(q,\tau \right)*\psi_{q}\left(\tau \right)$$

After EWT, the sub-signal with maximum spectrum energy is retained as interested echoes from target chest or abdomen, whereas the other sub-signals correspond to the reflection from other body parts and background clutters. Therefore, the signal superposition caused by body motions and background clutters is alleviated.

Considering that the body shift leads to the variation of $\tau_{1}$ and $\tau_{2}$, the maximum amplitude along the fast time of each retained signal is extracted as the vital signal. Due to motion interference, the waveform of obtained vital signal is distorted and polluted in various degrees. Moreover, the vital signal also contains multi-order harmonics of vital signs, which are overlapped on the frequency band. It is intractable to extract heart rate and breath rate by FFT directly. Improved EWT with Fourier segmentation refinement is proposed to further decompose chest and abdomen vital signals and extract vital signs-related sub-signals from two radars, respectively.

### 3.3. Vital Signs Sub-Signals Extraction Based on Improved EWT

The obtained vital signal is first smoothed by a Gaussian filter to mitigate signal distortion partly. Next, the filtered signal is normalized and subtracted by the signal average. With the EWT introduced in Section 3.2, the decomposition is guided by spectrum segmentation. It is essential to divide Fourier spectrum with appropriate frequency boundaries to obtain vital signs sub-signals, so that the refined frequency boundaries are constructed to tackle this challenge.

Assuming that the frequency band of heart rate is between $\left[f_{h_{0}},f_{h_{h}}\right]$, and the breath frequency band is between $\left[f_{b_{0}},f_{b_{h}}\right]$, the initial boundaries for the Fourier spectrum are set as $\left(f_{b_{0}},f_{b_{h}},f_{h_{0}},f_{h_{h}}\right)$. Moreover, based on the Fourier spectrum, initial boundaries are then adaptive fine-tuned to the frequencies corresponding to the nearest neighborhood minima, which are regarded as $\left(f_{h_{0}}^{{}^{\prime }},f_{h_{h}}^{{}^{\prime }},f_{b_{0}}^{{}^{\prime }},f_{b_{h}}^{{}^{\prime }}\right)$, respectively. Moreover, the refined frequency segments related to breath and heartbeat are obtained, which are between $\left[f_{h_{0}}^{{}^{\prime }},f_{h_{h}}^{{}^{\prime }}\right]$ and between $\left[f_{b_{0}}^{{}^{\prime }},f_{b_{h}}^{{}^{\prime }}\right]$, respectively.

Considering the overlapping of harmonics and residual motion noise in the vital signs bands, the obtained segments related to heartbeat and breath need to be further segmented. The segmentation strategy based on local maxima and minima described in Section 3.2 is applied on these segments, respectively. For chest vital signal, the maximum sub-segment numbers of refined breath and heartbeat segments are set as $N_{b_{c}}$ and $N_{h}$, respectively, so that the refined frequency boundaries are extended as $\left(f_{b_{0}}^{{}^{\prime }},f_{b_{1}}^{{}^{\prime }},\ldots ,f_{b_{U-1}}^{{}^{\prime }},f_{b_{h}}^{{}^{\prime }},f_{h_{0}}^{{}^{\prime }},f_{h_{1}}^{{}^{\prime }},\ldots ,f_{h_{V-1}}^{{}^{\prime }},f_{h_{h}}^{{}^{\prime }}\right),U\leq N_{b_{c}},V\leq N_{h}$. With the guidance of these boundaries, the EWT is applied on the chest vital signal. Moreover, a series of chest sub-signals are extracted, which are indicated as $\left\{{\left\{I_{j}^{b}\right\}}_{j=1}^{U},{\left\{I_{i}^{h}\right\}}_{i=1}^{V}\right\}$, where $I_{j}^{b}$ and $I_{i}^{h}$ denote the sub-signals extracted from breath and heartbeat band, respectively.

As for abdomen vital signal, only the refined breath segment is subdivided to further extract breath-related sub-signals. The maximum sub-segment number of the breath sub-spectrum is set as $N_{b_{a}}$. Moreover, the abdomen boundaries are obtained as $\left(f_{b_{0}}^{{}^{\prime }},f_{b_{1}}^{{}^{\prime }},\ldots ,f_{b_{W-1}}^{{}^{\prime }},f_{b_{h}}^{{}^{\prime }},f_{h_{0}}^{{}^{\prime }},f_{h_{h}}^{{}^{\prime }}\right),W\leq N_{b_{a}}$. A series of abdomen sub-signals are obtained as $\left\{{\left\{I_{k}^{b}\right\}}_{k=1}^{W},I_{o}\right\}$, where $I_{o}$ denotes the sub-signal in the heartbeat band.

According to Equations (3) and (4), the decomposed chest sub-signals consist of breath signal and its harmonics, heartbeat and its intermodulation signal with breath. Besides, there exists residual motion artifact related sub-signal. The abdomen sub-signals only contain breath-related sub-signals and motion artifact-related sub-signal. In order to extract vital signs under the interference from multi-order harmonics and residual motion artifact, cross-correlation functions are calculated among chest and abdomen sub-signals to estimate heartbeat and breath.

### 3.4. Cross-Correlation Functions Based Vital Signs Estimation

#### 3.4.1. Breath Estimation

Based on the breath correlation of chest and abdomen signals, the dependency is first evaluated among different decomposed sub-signals to assist breath signal selection from chest and abdomen sub-signals. Cross-correlation function provides the relational degrees of two signals at different times, which is applied to present sub-signals dependency. The breath cross-correlation function $R_{jk}\left(n\right)$ calculated on $I_{j}^{b}$ and $I_{k}^{b}$ is expressed as

(10) $$R_{jk}\left(n\right)=\frac{1}{D}\int_{0}^{D}I_{j}^{b}\left(t\right)I_{k}^{b}\left(t+n\right)dt,-D<n<D,$$

where *n* denotes the time shift on radar slow time, and *D* denotes the time span of the two sub-signals. The maximum value of $R_{jk}\left(n\right)$ is extracted as a relevance coefficient to indicate the degree of sub-signal relevance, which is denoted as $r_{jk}$. After calculating $R_{jk}\left(n\right)$ iteratively, the relevance coefficient matrix $C_{b}$ of breath is obtained and expressed as

(11) $$C_{b}=\left(\begin{array}{ccc} r_{11} & \ldots & r_{1W}\\ \vdots & \ddots & \vdots \\ r_{U1} & \cdots & r_{UW} \end{array}\right).$$

**Figure 4:**
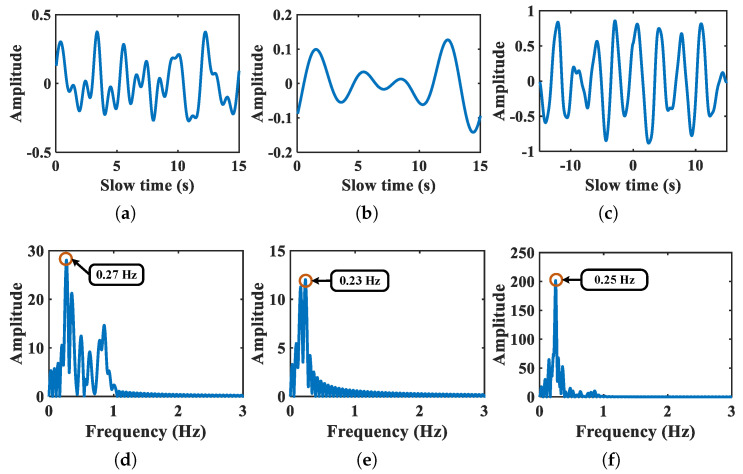
Breath sub-signals of chest and abdomen and corresponding breath cross-correlation function. Panels (**a**,**b**) denote the waveform of breath sub-signals of chest and abdomen, respectively. Panel (**c**) denotes the breath cross-correlation function. Panels (**d**–**f**) denote the corresponding Fourier spectrum, respectively.

Compared with residual motion artifact and breath harmonics, the fundamental breath signal presents a stable periodicity so that two breath sub-signals extracted from target chest and abdomen present stronger relevancy. Therefore, the maximum relevance coefficient of $C_{b}$ corresponds to the breath related sub-signals obtained from chest and abdomen, respectively. Figure 4 shows the waveform and frequency spectrum of selected breath sub-signals and corresponding cross-correlation function. The real breath rate of the shown example is 0.25 Hz. As is depicted in Figure 4a,b, two breath sub-signals are partly distorted under motion interference, resulting in the detected breath frequency with larger absolute errors, which are all 0.02 Hz. It is notable that the corresponding breath cross-correlation function in Figure 4c overcomes the effect of signal deformation and presents obvious periodicity related to breath vibration. Moreover, the absolute error of estimated breath frequency decreases to zero so that the breath frequency is extracted by the selected breath cross-correlation function.

#### 3.4.2. Heartbeat Estimation

Considering the limitation of radar detection view, the lower radar barely detects heartbeat vibration. The decomposed sub-signal working on heartbeat band obtained from target abdomen indicates residual interference signal, for example, motion artifact signal so that the heartbeat sub-signal of chest have little correlation with the sub-signal in the same band extracted from abdomen signal. The heartbeat cross-correlation function $R_{io}\left(n\right)$ is calculated iteratively on chest heartbeat-related sub-signals $I_{i}^{h}$ and corresponding abdomen sub-signal $I_{o}$ as Equation (10) is described. Moreover, the heartbeat relevance coefficient matrix $C_{h}$ is obtained as

(12) $$C_{h}=\left(r_{1o},r_{2o},\cdots ,r_{Vo}\right).$$

The maximum relevance coefficient of $C_{h}$ presents the sub-signal containing little heartbeat-related vibration, which is excluded from the chest sub-signals related to heartbeat. After that, the sub-signal with maximum signal energy is extracted as heartbeat signal $I^{h}$. Furthermore, the heart rate obtained by applying FFT on $I^{h}$.

## 4. Results and Discussion

### 4.1. Movement Interference Alleviation Performance Based on EWT

**Figure 5:**
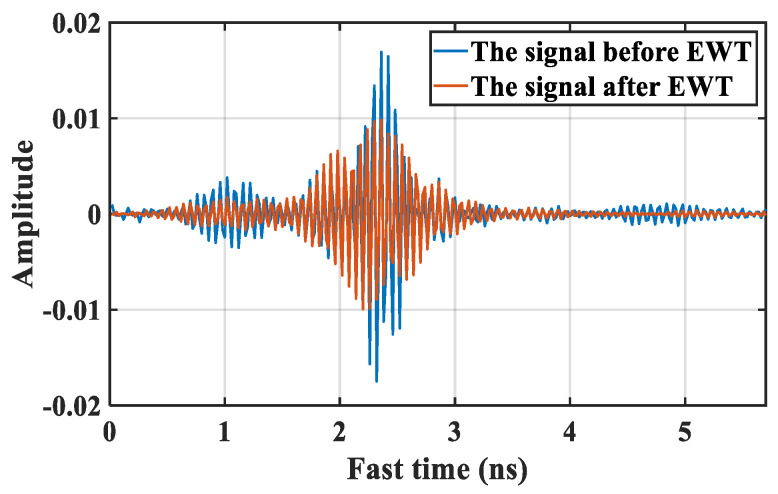
A signal of movement interference alleviation based on EWT.

Figure 5 shows a signal obtained from the upper radar as an example of movement interference alleviation based on EWT. The blue line indicates the signal before EWT. It is notable that there exists overlapping of target chest and other body parts echoes. Furthermore, the signal after interference mitigation is depicted by red line. The signal retained is reflected from target chest. The amplitude of the retained signal after EWT is decreased partly. It is because the motion artifacts overlapping with the chest signal are suppressed.

**Figure 6:**
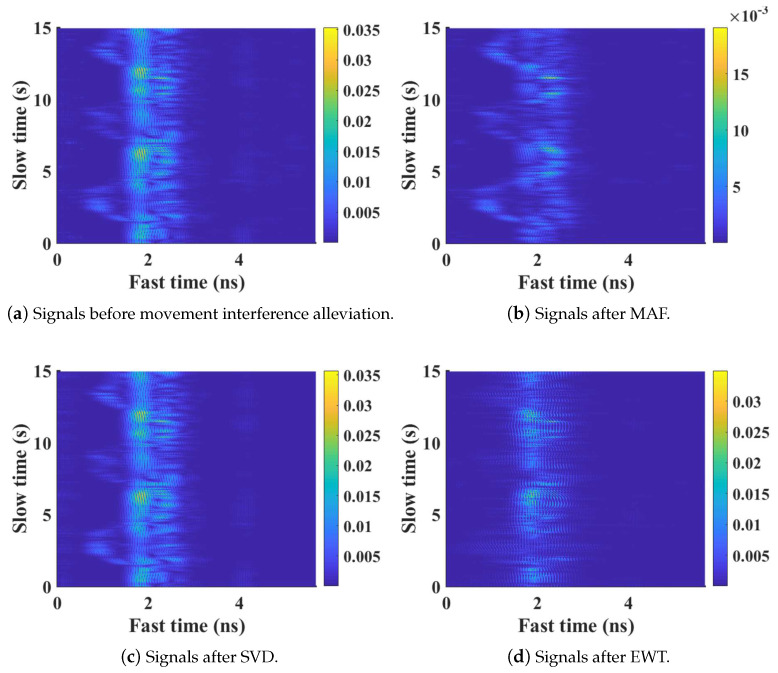
The performance of movement interference alleviation using different methods.

In order to further evaluate the performance of movement interference alleviation based on EWT, moving average filtering (MAF) [29], and Singular Value Decomposition (SVD) [43] are introduced as comparisons. Figure 6 presents a motion interference mitigation example based on different methods. The shown signals are collected in Exercise II situation by the upper radar. Figure 6a describes the target signals obtained by DC removal. It is notable that the signal reflected from target chest are superposed and obstructed by echoes from other body parts, which are mainly induced by the arm waving while the participant doing exercise. As is shown in Figure 6b,c, there still exists strong motion superposition after using comparison methods. After applying EWT, as is shown in Figure 6d, the arm movement interference is effectively suppressed, whereas the chest echoes are still retained. The effectiveness of movement interference alleviation based on EWT is demonstrated.

Furthermore, a signal-to-movement-interference ratio (SMIR) is introduced to analyze the performance of motion artifact reduction quantificationally, which presents an energy ratio of the signals reflected from target chest or abdomen under movement interference. SMIR is described as

(13) $$SMIR=\frac{E_{\tau_{1}\tau_{2}}}{E_{0}-E_{\tau_{1}\tau_{2}}},$$

where $E_{\tau_{1}\tau_{2}}$ denotes the summation of the signal energy between the interested fast time interval $\left[\tau_{1},\tau_{2}\right]$, and $E_{0}$ denotes the total signal energy. Based on the experimental setup, the distance range of chest-to-antenna distance and abdomen-to-antenna distance are between 0.5 m and 1 m. According to Equation (1), the corresponding fast time $\tau_{1}$ and $\tau_{2}$ are 3.3 ns and 6.7 ns, respectively. The results of these three methods applying on upper and lower radars are listed in Table 3.

As is shown in Table 3, the performance of the EWT-based method on SMIR is better than other two methods, which quantificationally demonstrates the validity of movement interference alleviation based on EWT. The SVD-based method obtains higher SMIR than MAF-based method. This is because the motion interference overlapping with body chest or abdomen is not suppressed effectively, resulting in larger signal energy in the interested fast time interval. After mitigating motion artifacts, the vital signals of target chest and abdomen are extracted from the maximum amplitude along the fast time of each retained signal.

### 4.2. Vital Signs Sub-Signals Extraction Results Based on EWT

**Figure 7:**
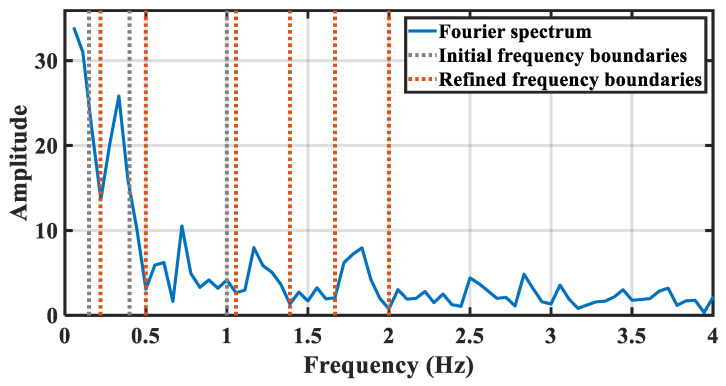
The description of chest spectrum segmentation refinement.

Figure 7 shows an example of spectrum segmentation refinement based on EWT. The blue line indicates the spectrum of a chest vital signal extracted from the upper radar. For a healthy adult, the heartbeat frequency band is set as 1–2 Hz, while the breath frequency band is set as 0.15–0.4 Hz. So that the initial frequency boundaries are set as 0.15 Hz, 0.4 Hz, 1 Hz, and 2 Hz, respectively, which are marked by the gray lines. Based on the initial frequency boundaries, the orange lines indicate the refined frequency boundaries including the adaptive fine-tuned boundaries, i.e, 0.22 Hz, 0.39 Hz, 1.06 Hz, and 2.06 Hz and sub-divided boundaries, i.e., 1.39 Hz, 1.67 Hz.

**Figure 8:**
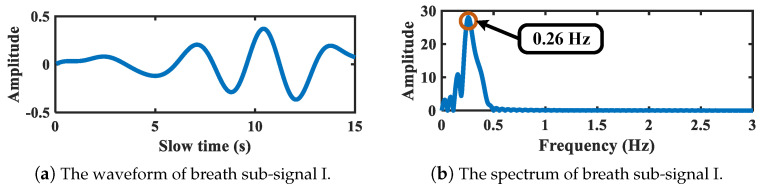
The breath-related sub-signal of target chest.

**Figure 9:**
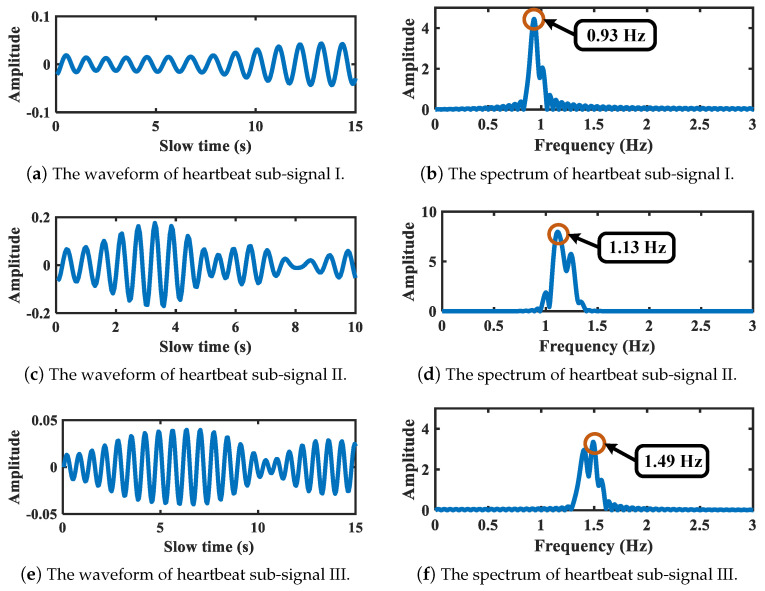
Heartbeat-related sub-signals of target chest.

After spectrum segmentation refinement, the EWT is applied. As is shown in Figure 8, the second segment corresponds to the breath-related sub-signal, while the fourth, fifth, and sixth segments correspond to heartbeat-related sub-signals. The waveforms and spectrums of decomposed breath-related sub-signals are shown in Figure 8. Considering that only one breath-related sub-signal is extracted, this sub-signal is regarded as a breath signal detected from the target chest. The vibration frequency is 0.26 Hz, corresponding to the peak of Fourier spectrum. Moreover, the waveforms and spectrums of decomposed heartbeat-related sub-signals are shown in Figure 9. Three sub-signals are obtained regarded as one heartbeat signal and two intermodulation signals of heartbeat and breath, with vibration frequency at 1.13 Hz, 0.93 Hz, and 1.49 Hz, respectively.

**Figure 10:**
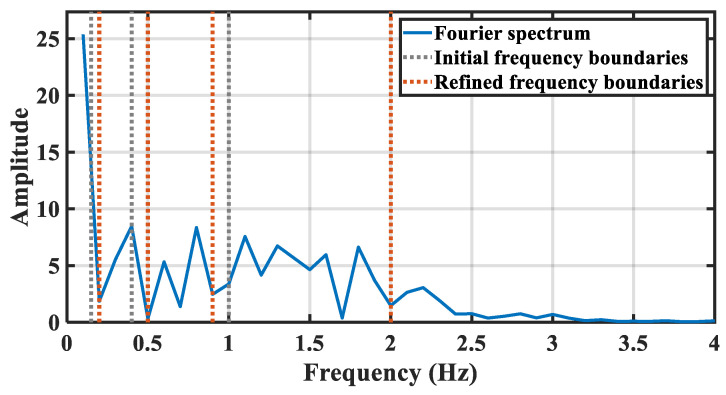
The description of abdomen spectrum segmentation refinement.

**Figure 11:**
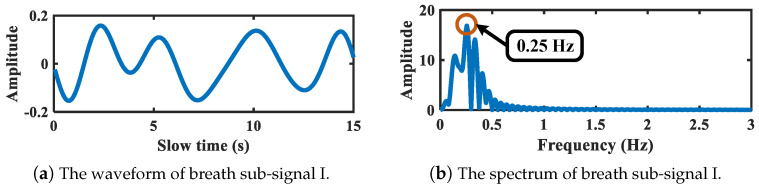
Breath-related sub-signal of target abdomen.

**Figure 12:**
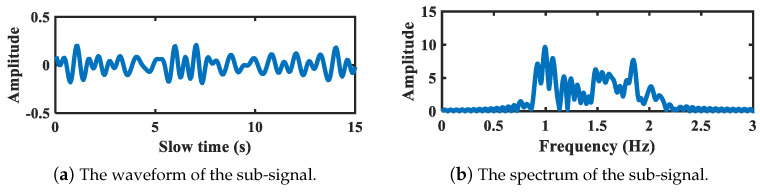
The sub-signal working on heartbeat band of target abdomen.

Figure 10 shows the refined spectrum segmentation result of the abdomen vital signal corresponding to above chest signal. As is depicted, after refinement, the frequency boundaries are set as 0.11 Hz, 0.44 Hz, 0.94 Hz, and 2 Hz, respectively. The breath-related segment can be further segmented if needed, and the segment corresponding to heartbeat band is not sub-divided anymore. The decomposed sub-signals are illustrated in Figure 11 and Figure 12. Figure 11 shows the breath sub-signal extracted from target abdomen with vibration frequency 0.25 Hz, whereas Figure 12 shows the sub-signal working on heartbeat band.

### 4.3. Evaluation on Vital Signs Estimation

The breath rate estimation absolute error $Er_{b}$ is introduced to evaluate the breath monitoring performance, which is expressed as

(14) $$Er_{b}=\mathrm{abs}\left(br_{e}-br_{t}\right),$$

where $br_{e}$ indicates the estimated breath rate, and $br_{t}$ indicates the collected true breath rate obtained from the oximeter.

The heart rate estimation accuracy $Acc_{h}$ is introduced to evaluate the heartbeat monitoring performance, which is expressed as

(15) $$Acc_{h}=\left(1-\frac{\mathrm{abs}\left(hr_{e}-hr_{t}\right)}{hr_{t}}\right)*100\%,$$

where $hr_{e}$ indicates the estimated heart rate, whereas $hr_{t}$ indicates the reference heart rate obtained from the oximeter.

The estimated vital signs of eight participants based on the proposed approach are listed in Table 4, which presents the performance evaluated on the heart rate accuracy and the breath rate absolute error. Avg and Std denote the average and the standard deviation of estimated results, respectively.

As is illustrated in Table 4, based on the proposed approach, the average breath rate estimation absolute error for eight participants is 2.3 with the standard deviation 1.7 respiration per minute (rpm), whereas the average heart rate estimation accuracy is 86.9% with the standard deviation 7.4%. The highest average heart rate estimation accuracy reaches 88.6% with the standard deviation 8.5% for participant 3. The listed results show the monitor robustness of the proposed approach on different people during PA.

In order to evaluate the performance on various activities, the experiments result of different activities are also obtained and listed in Table 5.

As is illustrated, the proposed algorithm achieves a good performance on five different activities. For all the activities, the heart rate estimation accuracy can reach 86.0% and the highest estimation accuracy is 88.1% for the back-and-forth body movement. The absolute error of breath rate is less than 2.6 rpm. Besides, during the experiment and signal processing, no extra expert intervention is conducted. The experimental results demonstrate the effectiveness of the proposed algorithm in practical usage on different activities.

In order to analyze the influence of slow-time duration of a radar sample on monitoring performance, the vital signs estimation performances based on radar samples sliced by slow-time windows with different lengths are described in Table 6. The average computation time of one-time radar sample processing are also obtained based on the experimental laptop with a Intel’s core i5 10th generation processor. By contrast, it is shown that the best vital signs estimation result is obtained based on the radar samples sliced by the selected 15 s window in this paper. Moreover, the shorter or longer windows are obviously negative to the performance of the proposed algorithm. Besides, it also have a moderate computing time when the radar samples are 15 s length.

In order to further evaluate the effectiveness of the proposed approach, three vital signs monitoring approaches are introduced as comparison, including Variational Mode Decomposition (VMD)-based method [44], Ensemble Empirical Mode Decomposition (EEMD) with Continuous Wavelet Transformation (CWT) method [14], and HEAR method [35]. Besides, the performance of the proposed approach applying on a single radar is also compared, which estimates vital signs based on the decomposed sub-signals with maximum signal energy in heartbeat and breath band, respectively.

#### 4.3.1. Comparisons on Breath Estimation

**Figure 13:**
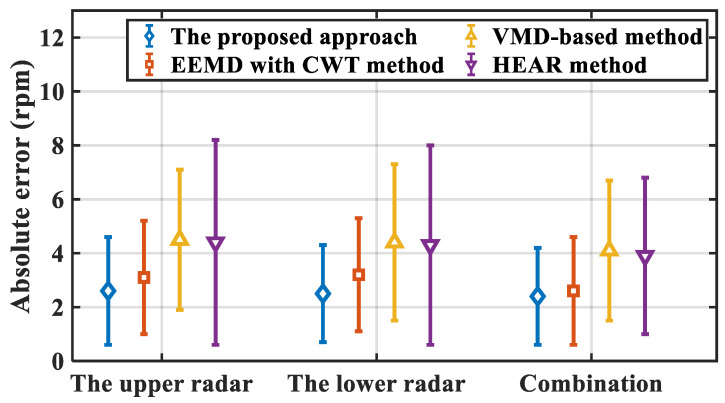
Breath estimation absolute error of different methods.

In order to verify the superiority of the proposed approach, three comparison methods mentioned above are applied on both upper and lower radar. Moreover, the average of estimated breath rates by two radars are also obtained. The results of breath estimation absolute error are performed in Figure 13.

As is illustrated, the marked points denote the averages of estimation absolute error, while the corresponding bars on both sides of the points denote the standard deviation of estimation absolute error for each method. It is notable that the proposed approach performs best based on the combination of two radar, the average absolute error of which is 2.3 rpm. The estimation absolute error is 2.6 rpm, 0.5 rpm, and 0.3 rpm less than the results of the other three methods, respectively. Furthermore, the result of the proposed approach applied on a single radar present superiority among these four methods. For upper and lower radar, the average absolute errors are 2.6 rpm and 2.5 rpm, respectively, both of which promote the performance of other three methods. Besides, the standard deviation of the proposed approach is smaller compared with other three methods, which presents the stability and robustness of estimation performance of the proposed approach.

The estimation errors of the other three methods are relatively large. For the VMD-based method and the EEWT with CWT method, the detection performances are strongly affected by untreated movement interference, while the complicated body motions hinder the breath estimation using HEAR. Moreover, compared with the estimation result based on two radars, the absolute error increases for each method based on a single radar. It is demonstrated that the superiority of the proposed approach on breath rate estimation with body movement by monitoring target chest and abdomen simultaneously.

#### 4.3.2. Comparison Results on Heartbeat Estimation

In order to evaluate the performance of heartbeat estimation based on the proposed approach, three aforementioned methods are introduced and applied on the upper radar as comparisons. The heartbeat estimation results of these methods are listed in Table 7.

As illustrated in Table 7, it is observed that the proposed approach combining two radar signals presents the best performance on heart rate estimation among all the methods, the average accuracy of which achieves 86.9%. Compared with the proposed approach applying on the upper radar, the average accuracy improves 5.3% using two radars simultaneously, which illustrates the superiority for heartbeat estimation using two radars simultaneously. Moreover, the accuracy of estimated heart rate obtained by the proposed approach is 6%, 6.9%, and 7.9% higher than the comparison methods, respectively. The performances of the other three methods are affected by various body movement during PA. Although HEAR method deals with movement interference cause by the variation of antenna-to-target distance, it is insufficient to handle various types of body movement interference during PA and extract the signal reflected from target chest. Therefore, the effectiveness of the proposed approach is verified in heartbeat monitoring.

### 4.4. Discussions and Future Works

It is feasible to exploit the proposed approach in real cases, such as working in the office, driving a car, or spinning bike in the gym. In these cases, although various random body movements happen most of the time, the monitored person always maintain the upper body straight, and the two radars can be placed facing to the chest and abdomen of monitored person. In the future works, the proposed approach will be improved and evaluated in these scenarios.

For some much more complex human activities in real cases, such as continuous jumping, rapid spinning, and squatting up and down, the induced motion artifacts severely contaminate and destroy the vital signals detected by the IR-UWB radars. In these complex scenarios, the proposed approach needs to be improved further to guarantee monitoring performance. Besides, there exist real scenarios with multiple moving targets in a wide range. A promising direction for such scenarios with multiple moving targets in a wide range is extending the proposed approach by using more IR-UWB radars to cover the entire monitoring area and obtain vital signs of multiple moving targets in the future works.

## 5. Conclusions

In this paper, a novel joint chest–abdomen cardiopulmonary signal approach is proposed to detect breath and heart rate simultaneously using two IR-UWB radars during PA. In order to deal with the signal superposition caused by various movement artifacts during PA, EWT is introduced to remove clutters and alleviate motion interference of received radar signals. Furthermore, the interested vital signals reflected from target chest and abdomen are extracted. Moreover, improved EWT with frequency boundaries refinement is applied to decomposed vital signal of each radar as breath and heartbeat related sub-signals. After that, based on the thoracoabdominal correlation, the cross-correlation function is calculated among sub-signals of chest and abdomen, and estimate heartbeat and breath. The proposed approach is evaluated on three kinds of PA and two general body movements. The average estimation accuracy of heart rate is 86.9%, whereas the average absolute error of breath rate is 2.3 rpm. The experimental results demonstrate the effectiveness and superiority of the proposed approach.

## Figures and Tables

**Table 1:** The details of body motions of five kinds of activities.

Activities	Description
Yoga	Move gently and switch among three yoga poses, including the Mountain Pose, the Warrior I, and the Warrior II. Stretch body and hold a few seconds at each yoga pose.
Exercise I	Keep the body upright, wave arms up and down from both sides of the body repeatedly.
Exercise II	Keep the body upright, wave arms up and down from the front of the body repeatedly.
Random body movement	Engage some daily activities in the detection area such as making a phone call, scratching head, adjusting glasses.
Back-and-forth body movement	Waggle the upper body back and forth repeatedly.

**Table 2:** Physical information of the participants.

Participant	1	2	3	4	5	6	7	8
Gender	Female	Female	Female	Female	Male	Male	Male	Male
Age (year)	24	22	23	24	23	24	28	23
Height (m)	1.63	1.67	1.60	1.58	1.87	1.70	1.74	1.86
Weight (kg)	54	51	53	52	83	52	68	95

**Table 3:** Comparisons on SMIR.

	SMIR after MAF	SMIR after SVD	SMIR after EWT
The upper radar	3.4	20.5	25.3
The lower radar	3.3	14.2	16.6

**Table 4:** Vital signs estimation results on different participants using the proposed approach.

Participant	Breath Rate Absolute Error		Heart Rate Accuracy	
	Avg (rpm)	Std (rpm)	Avg (%)	Std (%)
1	2.6	1.7	86.5	11.6
2	2.2	1.8	87.7	8.9
3	2.3	1.8	88.6	8.5
4	2.6	1.7	87.2	8.1
5	2.2	1.8	86.7	9.1
6	2.7	1.9	84.7	11.1
7	1.9	1.7	87.6	9.5
8	2.4	1.7	88.0	7.4
Average	2.3	1.8	86.9	9.8

**Table 5:** Vital signs estimation results on different activities using the proposed approach.

Activities	Breath Rate Absolute Error		Heart Rate Accuracy	
	Avg (rpm)	Std (rpm)	Avg (%)	Std (%)
Exercise I	2.1	1.8	86.3	9.7
Exercise II	2.2	1.8	86.0	10.8
Yoga	2.4	1.9	86.4	10.8
Random body movement	2.6	1.7	86.4	9.9
Back-and-forth body movement	2.6	2.0	88.1	9.0

**Table 6:** The estimation performances based on radar samples sliced by slow-time windows of different length.

	Breath Rate Absolute Error (rpm)	Heart Rate Accuracy (%)	Computation Time (s)
7-s window	2.8	84.4	0.43
10-s window	2.9	85.6	0.53
15-s window	2.3	86.9	0.79
18-s window	2.5	86.6	0.92

**Table 7:** Comparisons on heartbeat estimation among different methods.

	Approach	Accuracy
	HEAR method	79.0%
The upper radar	VMD-based method	80.0%
	EEMD with CWT method	80.9%
	The proposed approach	81.6%
Combinaton	The proposed approach	86.9%

## Data Availability

The collected radar data and the signal processing code are available at https://github.com/jocelynZXY/RadarDataforCSBHRD accessed on 12 August 2021.
